# Sex-specific nicotine sensitization and imprinting of self-administration in rats inform GWAS findings on human addiction phenotypes

**DOI:** 10.1038/s41386-021-01027-0

**Published:** 2021-05-18

**Authors:** Alena Kozlova, Robert R. Butler, Siwei Zhang, Thomas Ujas, Hanwen Zhang, Stephan Steidl, Alan R. Sanders, Zhiping P. Pang, Paul Vezina, Jubao Duan

**Affiliations:** 1 Center for Psychiatric Genetics, NorthShore University HealthSystem, Evanston, IL USA; 2 Department of Psychiatry and Behavioral Neurosciences, University of Chicago, Chicago, IL USA; 3 Department of Psychology, Loyola University Chicago, Chicago, IL USA; 4 Department of Neuroscience and Cell Biology and Child Health Institute of New Jersey, Rutgers University, New Brunswick, NJ USA

**Keywords:** Gene expression profiling, Addiction, Imprinting

## Abstract

Repeated nicotine exposure leads to sensitization (SST) and enhances self-administration (SA) in rodents. However, the molecular basis of nicotine SST and SA and their biological relevance to the mounting genome-wide association study (GWAS) loci of human addictive behaviors are poorly understood. Considering a gateway drug role of nicotine, we modeled nicotine SST and SA in F1 progeny of inbred rats (F344/BN) and conducted integrative genomics analyses. We unexpectedly observed male-specific nicotine SST and a parental effect of SA only present in paternal F344 crosses. Transcriptional profiling in the ventral tegmental area (VTA) and nucleus accumbens (NAc) core and shell further revealed sex- and brain region-specific transcriptomic signatures of SST and SA. We found that genes associated with SST and SA were enriched for those related to synaptic processes, myelin sheath, and tobacco use disorder or chemdependency. Interestingly, SST-associated genes were often downregulated in male VTA but upregulated in female VTA, and strongly enriched for smoking GWAS risk variants, possibly explaining the male-specific SST. For SA, we found widespread region-specific allelic imbalance of expression (AIE), of which genes showing AIE bias toward paternal F344 alleles in NAc core were strongly enriched for SA-associated genes and for GWAS risk variants of smoking initiation, likely contributing to the parental effect of SA. Our study suggests a mechanistic link between transcriptional changes underlying the NIC SST and SA and human nicotine addiction, providing a resource for understanding the neurobiology basis of the GWAS findings on human smoking and other addictive phenotypes.

## Introduction

Among over 4800 chemical compounds in tobacco, nicotine (NIC) determines the addictive nature of smoking [1]. NIC is also a possible gateway “drug” for other substances of abuse [2, 3]. Genome-wide association studies (GWAS) of cigarette smoking have identified a plethora of genetic loci with single nucleotide polymorphisms (SNPs) associated with smoking phenotypes [4–8]. A recent GWAS meta-analysis with ~1.2 million individuals identifies 406 risk loci for smoking initiation (SIn; a binary indicating whether an individual had ever smoked regularly), cessation (SCe; a binary for quitting smoking), cigarettes per day (CPD), and age of initiation (AOI; age of initiation of regular smoking) [9]. However, each risk locus often spans many equivalently associated genetic variants and genes, leaving the exact risk genes and molecular mechanisms for many GWAS risk loci undetermined.

Much of our knowledge of NIC function stems from studying NIC addiction in rodents. A key process of developing NIC-related addictive behaviors in rodents is NIC sensitization (SST). When NIC is repeatedly administered, its effects are enhanced so that its re-exposure later produces greater locomotor activity [2, 3]. NIC SST may have relevance to the initiation, maintenance, and escalation of NIC use that is characteristic of the transition from casual experimentation of NIC or other drugs to craving and abuse in humans [2]. Although NIC acetylcholine receptors expressed in the ventral tegmental area (VTA) are known to mediate the effect of NIC on dopaminergic release in the nucleus accumbens (NAc) and locomotor activity [10–12], the SST-associated transcriptomic changes and their relevance to human smoking traits remain elusive.

A similar knowledge gap also exists for another addiction model, NIC self-administration (SA) [1]. Intravenous SA of NIC can be used to determine how motivated animals are to obtain NIC, as indexed by the number of lever presses they are willing to invest in order to earn an infusion [2, 13]. However, it remains largely unknown how the gene expression changes associated with SA are relevant to genetic findings of human smoking traits and other addictive behaviors. Furthermore, the molecular link between NIC SA and SST is unclear, highlighting the need of parallel transcriptomic profiling of NIC SST and SA in relevant brain regions.

Previous transcriptomic studies mostly analyze the acute effects of NIC rather than the molecular underpinning of NIC addiction, showing NIC-induced expression related to G-protein-coupled-receptor signaling [14], glycerolipid metabolism [15], and Hedgehog/Notch pathways [16]. Here, we identified transcriptomic signatures of sex-specific NIC SST and SA in addiction-relevant brain regions: VTA, NAc core and shell. For the observed parental effect (or imprinting) of NIC SA, we performed allelic imbalance of expression (AIE) analyses to identify genes that may be responsible for this effect.

## Materials and methods

### Animals and crosses

F1 progeny were generated by crossing multiple breeding pairs (F344/NHsd[F344] and BN/RijHsd[BN]) purchased from Envigo. F1 rats of the initial cross (F1i), F344 father/BN mother (subgroup A), and of the reciprocal cross (F1r), BN father/F344 mother (subgroup B), were evenly distributed in all experimental groups. The breeding was carried out at the University of Chicago Animal Resources Center. All rats were single housed for at least one week before conducting any procedures. All experiments were performed during the dark phase of the light cycle according to an approved Institutional Animal Care and Use Committee protocol.

### Behavioral tests

Determination of specific aspects of the experimental procedures described below (NIC concentrations, number of NIC injections, and interval between injections and testing) was based on our previous reports [2, 12, 13, 17].

For SST, F1 males (*n* = 5 for the first and second groups, *n* = 6 for the third group) and females (*n* = 7 for all three groups) were randomly assigned to three groups and tested in early adulthood (~250 g). Rats in the first group were administered 0.1 mg/kg of NIC, those in the second 0.4 mg/kg, and those in the third saline (SAL;1.0 ml/kg). Injections were given once daily for 4 days. On days 1 and 4, rats were placed in an open field immediately after the injection and their locomotion measured for 2 h. Rats were then left undisturbed for 2 weeks after which they were tested for locomotor SST. On this test, all F1 rats were administered one injection of NIC (0.4 mg/kg), immediately placed in the open fields and their locomotion measured for 2 h. For transcriptomic analysis, F1 males (*n* = 16) and females (*n* = 16) were each divided into two groups, each administered with 0.4 mg/kg NIC or SAL every day for 4 days. Brain tissues (~20 mg) from NAc core, NAc shell, and VTA regions were harvested 2 weeks later [18].

For NIC SA, F1 males (*n* = 8) were prepared with an IV catheter and allowed to self-administer NIC (30 μg/kg/infusion) on each of 6 daily 2-h SA sessions. Each SA chamber contained one lever, presses on which delivered a NIC infusion on a FR1 schedule of reinforcement. The number of actively self-administered infusions was recorded. A maximum of ten infusions (active + passive) was allowed per session. As a control, a non-catheterized group of no-NIC SA rats was also tested (*n* = 8). All remaining apparatus configurations and procedures were as described in [13]. Brain tissues were harvested 3 days following the last session.

### Brain tissue harvesting and homogenization

Brain tissues were hand-dissected using procedures described in Singer et al. [18] with the additional differentiation of the NAc core and shell illustrated in Fig. S1 [19]. Three hundred fifty microliters of cold lysis buffer was added to the brain tissue and sonicated (5 rounds: 3 s—pulse, 12 s—pause, amp 7). Tissue was triturated ten times through a 26-gauge needle (VWR, BD305111) and passed through Qiagen shredder (Qiagen, 79654) for 2 min at 14,000 × rpm at 4 °C.

### RNA isolation, sequencing (RNA-seq), and quantitative PCR (qPCR)

Total RNA was isolated from brain tissues using TRIzol LS reagent (Life technologies). The RNA sample was then cleaned with RNeasy MinElute Cleanup Kit (Qiagen), and subjected to RNA-seq at the University of Minnesota Genomic Center (NovaSeq S2 2 × 50 bp, >32M PE reads per library). qPCR confirmation was performed using TaqMan Universal PCR Master Mix and gene-specific FAM-labeled TaqMan probes (ThermoFisher) on a Roche 480 II instrument. Relative gene expression was calculated as described [20] with *GAPDH* as control.

### Transcriptomic analysis

RNA-seq reads were trimmed with fastp 0.20.0 [21] using −3, −5, and −1 20 settings, and mapped to Rnor version 6 [22] using salmon 0.12.0 [23] (71–77% mapped reads). Transcript abundance was calculated with recommended settings for PE reads and collapsed to gene counts using tximport 1.14.0[24]. A total of 16,069 genes and 16,063 genes had detectable expression (normalized reads greater than 10 in at least 3 samples) for the SST and SA experiments, respectively. Principal component analysis (PCA) and differential expression (DE) analysis were performed by using DEseq2 1.26.0 [25], utilizing the variance stabilizing transformation of DESeq normalized counts [26].

### Allelic imbalance of expression (AIE) analysis

The reads of all samples for each F1 type and tissue sample were merged and mapped to Rnor version 6 [22] using STAR 2.6.1d [27]. SNPs were called via GATK 4.1.4.1 [28]. To mitigate potential mapping bias to reference allele, we generated a masked fasta reference sequence with BEDTools 2.27.1 [29], which was then used for re-mapping. The resulting bam files were run through GATK HaplotypeCaller to generate a multi-sample all-calls vcf file. Bi-allelic SNPs were filtered for a minimum depth of 20 reads (≥2 for each allele). Allelic depths in A and B groups were summed across samples, and flagged as imbalanced (AIE) by binomial test (FDR < 0.05). AIE difference between subgroups A and B was estimated by a two-sample proportion test (FDR < 0.05, two-tailed test), requiring difference of reference-allele (BN copy) fraction to be >0.1.

### Gene set enrichment analysis (GSEA)

Gene sets were derived from Liu et al. [9] for AOI, smoking initiation (SIn), smoking cessation (SCe), CPD, and drinks per week, which were then tested for enrichment of SST and SA DE gene by Fisher’s exact test. Human orthologs of SST and SA genes were used in DAVID 6.8 [30] to test for enrichment of Genetic Association Database (GAD) disease phenotypes [31], KEGG Pathways [32], and Gene Ontology (GO) enrichments. STRING 11.0 protein interaction network analysis [33] was conducted on the subsets of overlapping genes between conditions.

### GWAS enrichment test using MAGMA

MAGMA 1.07b [34] enrichment of GWAS disease risk loci for SST and SA gene sets was done as described [35]. Summary statistics were from GWAS data sets of NIC phenotypes [9] and body mass index [36]. GWAS SNPs were limited to minor allele frequency >0.01 with a minimum INFO score >0.9.

### Statistical analyses

Behavioral data were analyzed by between and between-within analyses of variance (ANOVA) followed by post hoc Scheffé comparisons using the IBM SPSS Statistics module. The statistical analyses in DE test and gene set enrichment analyses were indicated separately in each section.

## Results

### Sex-specific NIC SST and imprinting of NIC SA in F344/BN F1 rats

**Fig. 1:**
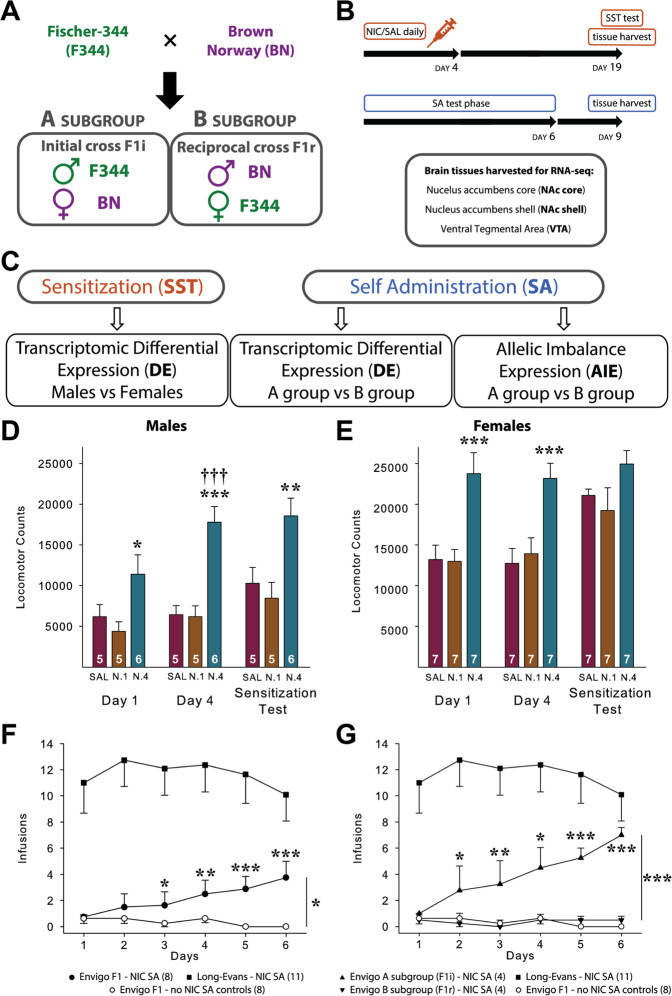
A schematic experimental design of NIC sensitization (SST) and self-administration (SA) and the behavioral tests. **A** Genetically identical and heterozygous F1 progeny of two inbred strains (F344 and BN) from both initial (F1i) and reciprocal (F1r) crosses were used. **B** The paradigm of NIC treatment for SST and SA tests. For SST transcriptomic analysis, brain tissues from the NAc core, shell, and VTA were harvested without testing SST at day 19 to avoid assaying the acute effect of NIC. **C** Genomics analyses for NIC SST and SA. **D**, **E** F1 males (*n* = 5–6/group) and F1 females (*n* = 7/group) were administered NIC (0.1 [N.1] or 0.4 [N.4] mg/kg; base, IP) or SAL daily for 4 days and tested for SST 2 weeks later. Data are mean (+SEM) of 2-h total locomotor counts obtained on days 1 and 4, and on the test for SST when all rats were administered NIC (0.4 mg/kg). **D** Males showed a dose-dependent increase in NIC-induced locomotion and NIC SST. ANOVA of the day 1 and day 4 results in the males revealed significant effects of the group [F_2,13_ = 10.43 (*p* < 0.01)], days [F_1,13_ = 15.55 (*p* < 0.01)], and a significant group × days interaction [F_2,13_ = 7.11 (*p* < 0.01)]. ANOVA of the test for sensitization results in these rats showed a significant group effect [F_2,13_ = 7.25 (*p* < 0.01)]. The denoted *p*-values were from post hoc Scheffé comparisons: *p < 0.05, **p < 0.01, ***p < 0.001, N.4 vs two other groups at indicated days. ^†††^*p* < 0.001, day 4 vs day 1 in N.4. **E** Females showed a dose-dependent increase in NIC-induced locomotion but did not exhibit NIC SST. ANOVA yielded only a significant effect of groups [F_2,18_ = 17.74 (*p* < 0.001)] for the exposure day 1 and 4 results in females. The denoted *p* values were from post hoc Scheffé comparisons: ***p < 0.001, N.4 vs two other groups at indicated days. **F** F1s with access to NIC as a group self-administered the drug significantly more than the non-NIC controls but much less than outbred Long-Evans rats. ANOVA of the results obtained in the two Envigo groups with and without access to NIC revealed a significant effect of groups [F_1,14_ = 9.35 (*p* < 0.05)] and a significant group × days interaction [F_5,70_ = 4.17 (*p* < 0.01)], with post Scheffé comparisons showing a progressively increasing and significantly higher intake in the rats with access to NIC starting on day 3 of SA (*p* < 0.05 − 0.001). **G** When the F1s with access to NIC were divided by type of reciprocal cross [F344 father/BN mother F1s (subgroup A) and F344 mother/ BN father F1s (subgroup B)] and the results reanalyzed, subgroup A F1 rats showed more inclined NIC SA that approached levels seen in the Long-Evans outbred rats by day 6. The ANOVA revealed significant effects of groups [F_2,13_ = 20.35 (*p* < 0.001)], days [F_5,65_ = 5.16 (*p* < 0.001)], and a significant groups × days interaction [F_10,65_ = 6.51 (*p* < 0.001)], with post hoc Scheffé comparisons showing a progressively increasing and significantly higher intake only in subgroup A relative to the other two groups starting on day 3: *p < 0.05, **p < 0.01, ***p < 0.001. Data in (**F**, **G**) are the group mean (±SEM) number of infusions rats self-administered. Data for the Long-Evans outbred rats are from [13].

We modeled NIC SST and SA using F1 progeny of two inbred Envigo strains (Fischer-344 [F344] and Brown Norway [BN]) (Fig. 1A, B). Both initial (F1i; subgroup A) and reciprocal crosses (F1r; subgroup B) were used to examine possible parent-of-origin effects[37–39]. The F1 rats of two inbred strains were all heterozygous, making them informative for AIE analysis to assess parental effects (Fig. 1).

NIC SST was tested separately in F1 males (*n* = 5–6/group) and females (*n* = 7/group) (see “Materials and methods”). We found that 0.4 mg/kg of NIC increased locomotor activity (vs. SAL and 0.1 mg/kg NIC) in both male and female rats on days 1 and 4. However, only males exhibited NIC SST 2 weeks later (Fig. 1D, E). The locomotor response of females was greater than that of the males throughout testing, but it did not show significant SST. Analysis of the time course data from the 2-h SST test showed that the lack of SST in females was not due to a ceiling effect (Fig. 1D, E) as levels of locomotion were far from maximal throughout the test. While levels of gonadal hormones during the estrous cycle have been suggested to contribute to stimulant SST in outbred females [40, 41], it is unlikely that estrous phase contributed in the F1 females in the present experiments as they did not show SST and did not show more variability than males in locomotor activity [42]. Thus, F344/BN F1 rats are responsive to NIC exposure, which has a long-lasting effect that leads to male-specific NIC SST.

Because only males show SST, we modeled SA in male F1 rats (see “Materials and methods”). The Envigo F1s as a group emitted more lever presses for NIC than the non-catheterized no-NIC SA controls, but much less than outbred Long-Evans rats (Fig. 1F; from [13] for illustration). However, when the data were reanalyzed by dividing the F1s by the type of reciprocal cross (i.e., A and B subgroups; Fig. 1A), we surprisingly found subgroup A, but not B, were more inclined to self-administer NIC (Fig. 1G). The NIC SA observed in subgroup A approached levels of Long-Evans rats (Fig. 1G). These results indicate that F344/BN F1 rats self-administer NIC with a strong parental effect.

### VTA region shows strongest transcriptomic relevance to NIC SST

**Fig. 2:**
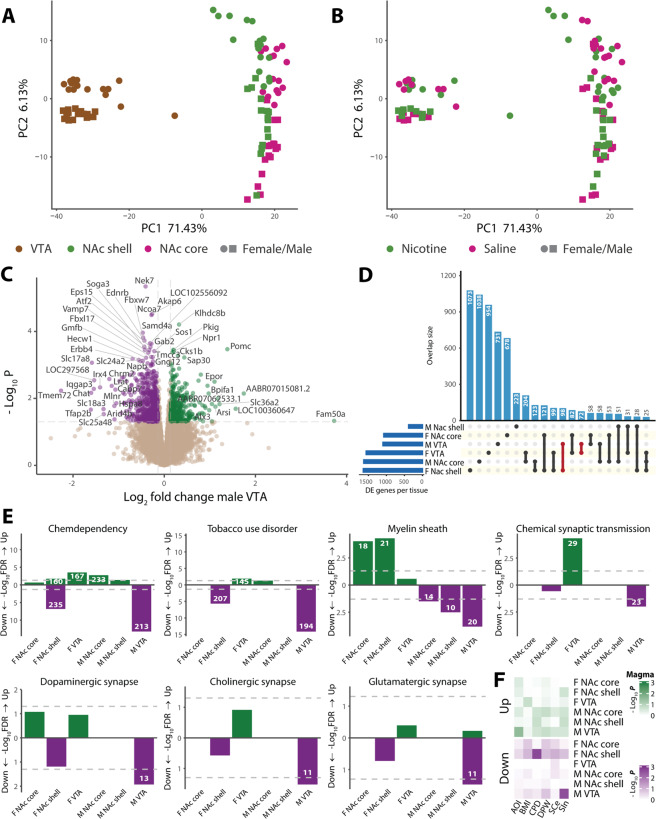
Transcriptomic analysis of NIC sensitization (SST). **A**, **B** Principal component analysis (PCA) of the top 500 differentially expressed (DE) genes in response to SST colored by (**A**) brain region, and by (**B**) NIC treatment; NAc nucleus accumbens, VTA ventral tegmental area. **C** Volcano plot of DE genes in the male VTA. **D** Upset plot of genes that are DE in different brain regions, with a nominal *p* < 0.05. Overlaps with *n* ≥ 25 are shown in vertical bars, while absolute DE gene counts for each tissue are represented in horizontal bars in the lower left. **E** DAVID gene set enrichment analysis of DE genes in each brain region, examining GAD diseases and disease classes, Online Mendelian Inheritance in Man (OMIM) diseases, KEGG pathways and GO terms. FDR-significant gene sets include the number of class genes (inset in the bar). **F** MAGMA enrichment analysis of genes harboring GWAS risk variants of five addiction phenotypes among DE genes in each brain region. Gene interval—100 kb window around the gene. AOI age of initiation, BMI body mass index, CPD cigarettes per day, DPW drinks per week, SCe smoking cessation, SIn smoking initiation; Up upregulated genes, Down downregulated genes, M male, F female.

With transcriptomic data of NIC SST, we first performed PCA to confirm the expected clear brain region-specific clustering (Fig. 2A), which was also confirmed by the expression profiles of genes related to NIC function (Fig. S2). RNA-seq samples were also well-separated by sex (Fig. 2A, B). We then identified DE genes in each brain region between NIC-treated and SAL groups. We found that the expression fold changes associated with NIC SST were small (<2-fold) (Fig. 2C and Fig. S3), consistent with a polygenic nature of NIC addiction. With a relaxed statistical cut-off (*p* < 0.05), we found 1629, 386, and 1097 DE genes in male NAc core, shell, and VTA (Fig. 2C, Table S1). Females showed a comparable number of DE genes (Table S2 and Fig. S3). The expression changes for some selected genes were confirmed by qPCR (Fig. S4). These genes were randomly selected from a list of top-ranking upregulated (Klhdc8b and Rev3l) or downregulated (Fbxl17, Ctnna2, Fam168a, and Cttnbp2) genes associated with SST in VTA or with SA in NAc core, which are also within smoking GWAS risk loci (Table S3). The overlaps of DE genes between brain regions and between sexes were small (Fig. 2D and Fig. S3), indicating region/sex-specific regulation.

To determine regions relevant to NIC SST, we first performed GSEA for DE genes using DAVID 6.8 [30, 43]. The downregulated genes in male VTA showed strong enrichment for genes related to tobacco use disorder and chemdependency, as well as myelin sheath, chemical synaptic transmission, and nervous system development (Fig. 2E and Fig. S5). In contrast, the upregulated genes in the VTA showed no (for males) or a lesser (for females) enrichment of genes related to brain function (Fig. 2E and Fig. S5). To further ascertain the regional transcriptional relevance to NIC addiction, we used MAGMA [34] to analyze the enrichment of SST-associated DE genes for GWAS associations with smoking and alcohol use disorders (AUD) [9]. We found that downregulated genes in the male VTA and female NAc shell showed enrichment (*p* < 0.003; FDR < 0.05) for SIn and CPD, respectively (Fig. 2F). Approximately thirty percent of the top-ranking DE genes (*n* = 100) in the male VTA are associated with smoking GWAS phenotypes (*p* < 5 × 10^−8^) (Table S3). These results demonstrate a predominant role of VTA genes in mediating male-specific NIC SST that is relevant to human smoking phenotypes.

### Opposing transcriptional changes in the VTA partially explain sex-specific NIC SST

**Fig. 3:**
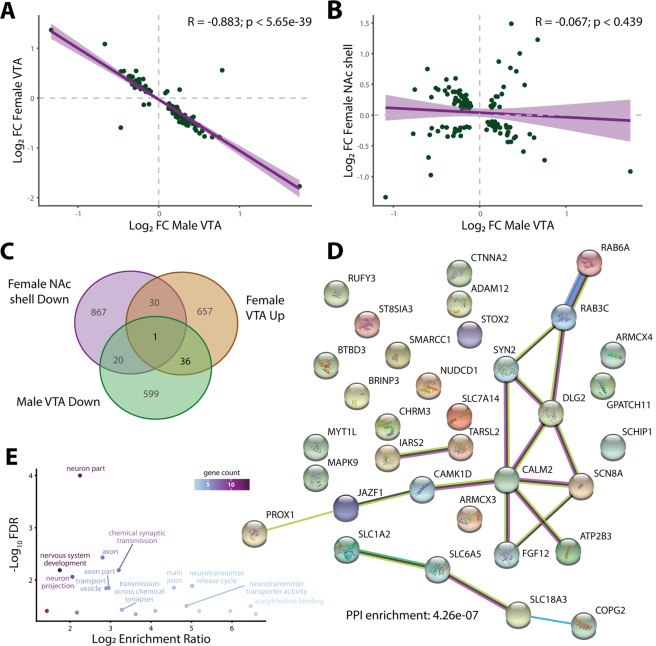
Comparison of sensitization (SST)-associated differential expression (DE) between males and females. **A** Fold change comparison of genes with nominally significant (*p* < 0.05) DE in both ventral tegmental areas (VTAs) between males and females. **B** Fold change comparison of genes with nominally significant (*p* < 0.05) DE in the male VTA and female NAc shell. **C** Venn diagram of nominally significant (*p* < 0.05) DE genes in directions/regions enriched for addiction phenotypes. Note: male VTA downregulated, female VTA upregulated, and female NAc shell downregulated genes are most relevant to NIC addiction based on DAVID and MAGMA enrichment analyses (Fig. 3, Figs. S2, S5). **D** STRING analysis of human orthologs of 37 genes with opposite direction of DE (*p* < 0.05) in male (down) and female (up) VTAs. Number of nodes: 34, number of edges: 18, average node degree: 1.06, avg. local clustering coefficient: 0.305, expected number of edges: 4, PPI enrichment *p* < 4.3 × 10^-7^. **E** Ontological enrichments from the STRING analysis, colored by gene count in each term.

To understand the molecular basis of the sex-specific SST (Fig. 1D, E), we examined the correlation of NIC-induced expression between male and female F1 rats. For this, we observed a negative correlation (*R* = −0.25, *p* < 1.48 × 10^−218^) of global NIC-induced expression changes in the VTA and NAc shell, but not in the NAc core (Fig. S6A–C). A stronger negative correlation was found for the subset of DE genes in male and female VTAs (*R* = −0.88, *p* < 5.65 × 10^−39^; *n* = 115, Table S4, Fig. 3A). On the contrary, no significant correlation of expression was found between male VTA and female NAc shell (Fig. 3B). The strong negative correlation of DE genes between male and female VTA was not due to their baseline (i.e., SAL-treated) expression, which showed strong positive correlation (*R* = 0.99, *p* < 6.42 × 10^−145^) (Fig. S6D). Thus, the overlapping DE genes in male and female VTAs with opposing expression changes likely contribute to the male-specific NIC SST.

The analyses of functional properties of the 37 negatively correlated male VTA-down/female VTA-up genes (Fig. 3A, C) further strengthen their importance in male-specific SST. Five out of the 37 overlapping genes (13.5%) encompass SNPs associated with CPD and/or Sin (Table S4) (vs. 4.1% among all expressed genes), representing 3.3-fold enrichment (Fisher’s exact test, *p* < 0.02). STRING [44] analysis of these 37 genes showed an enrichment for protein–protein interaction (PPI) (Fig. 3D) notably with Slc18a3, a vesicular acetylcholine transporter, as a hub gene, and for neuronal GO terms (Fig. 3E). The likely role of the 37 negatively correlated genes (Fig. 3C) in sex-specific SST was further supported by their enrichment for chromosome X (ChrX) genes (3.2-fold; Fisher’s exact test, *p* < 0.03): there are 4 ChrX genes (*LOC100910130*, *Atp2b3*, *Armcx4*, *Armcx3*) (10.8%; vs. 3.4% of all non-overlapping genes) (Table S4) and *Atp2b3*, which is also part of the enriched PPI network (Fig. 3D), encodes a calcium pump responsible for sex-specific pain responsiveness in mice [45, 46]. Altogether, these results suggest that the NIC-induced opposing transcriptional changes in male/female VTA may mechanistically contribute to the male-specific NIC SST.

### NAc core and VTA show strong transcriptomic relevance to NIC SA

**Fig. 4:**
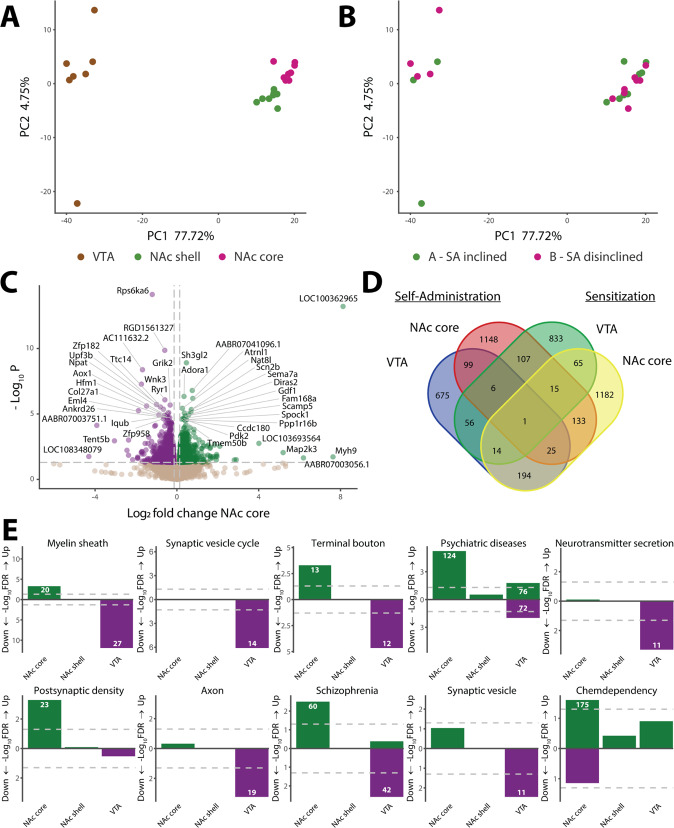
Transcriptomic analysis of NIC self-administration (SA). To mitigate the possible confounding effect of NIC exposure, all rats received ten infusions of NIC per session by providing a sufficient number of passively administered priming infusions (see “Materials and methods”), and brain tissues were harvested 3 days following the last SA session. **A**, **B** PCA analysis of the top 500 differentially expressed (DE) genes in response to SA colored by (**A**) brain region and by (**B**) F1 cross subgroup (which experimentally was also the SA inclined/disinclined distinction); NAc nucleus accumbens, VTA ventral tegmental area. **C** Volcano plot of 15,443 DE genes in the male NAc core. **D** Venn diagram of the DE (*p* < 0.05) genes in different brain regions. NAc core and VTA of SA experiments are compared to NAc core and VTA of SST experiments. **E** DAVID gene set enrichment analysis of DE genes in each brain region, examining GAD diseases and disease classes, OMIM diseases, KEGG pathways, and GO terms. FDR-significant gene sets include the number of genes (inset in the bar). Shown on the *y*-axis are enrichment significance (−Log_10_ FDR), Up upregulated genes, Down downregulated genes.

Leveraging our unexpected observation that only the subgroup A rats (F344 as paternal strain) (Fig. 1A) showed inclination for SA (Fig. 1G), we analyzed SA-associated DE genes by directly comparing the A vs. B subgroups. We found a clear transcriptomic separation of three brain regions (Fig. 4A), but not the A and B subgroups (Fig. 4B). Out of 16,063 expressed genes, we found 1534, 387, and 1070 DE genes (*p* < 0.05) in NAc core, shell, and VTA, respectively (Table S5). Most DE genes showed small fold-changes (<2-fold) (Fig. 4C and Fig. S7A), and very few DE genes overlapped between brain regions (Fig. 4D; Fig. S7B), suggesting robust region-specific effect.

To identify the brain regions transcriptionally relevant to NIC SA, we performed GSEA of the DE genes. We found that both the NAc core and the VTA were biologically relevant (Fig. 4E and Fig. S8): the upregulated genes in NAc core showed the strongest enrichments for psychiatric diseases, chemdependency, and GO terms such as neurotransmitter secretion, postsynaptic density and myelin sheath; the downregulated genes in VTA were also enriched for psychiatric diseases and GO terms related to neuronal function such as myelin sheaths, axon and neuron projection, neurotransmitter release, and synaptic vesicle. MAGMA [34] GWAS enrichment analysis further showed VTA-downregulated genes are enriched (*p* < 0.008) for those associated with AOI (not shown). Among the 100 top-ranking DE genes in the NAc core, 8 were associated with SIn (Table S3). These results suggest that transcriptional changes in the NAc core and VTA are relevant to NIC SA.

### NIC SA and SST are molecularly linked processes involving neurogenesis and myelin sheath

Transcriptomic profiling of NIC SST and SA with the same set of male F1 rats enabled us to examine the possible molecular links between the two processes. Despite the small overlap of the DE genes associated with NIC SST and SA (Fig. 4D), the overlapping genes between the most relevant regions for SST (VTA) and SA (NAc core) showed a negative correlation of DE (*R* = −0.50, *p* < 2.24 × 10^−9^) (Fig. S9A). The negative correlation was largely driven by SA-NAc-core-upregulated and SST-VTA-downregulated genes (Fig. S9A–C and Table S6). For VTA, we observed a strong positive correlation of DE genes for SST and SA (*R* = 0.66 and *p* < 1.2 × 10^−10^) when a single outlier gene (*Tmem72*) was removed (Fig. S9B and Table S7). STRING [44] analyses of both sets of overlapping genes showed significant enrichments for PPI network (Fig. S9D, E), and for GO terms related to neurogenesis and myelin sheath (Fig. S9F, G, Tables S8, and S9). Of the 97 overlapping genes (SA NAc core-up and SST VTA-down), 10 were associated with smoking phenotypes (mostly SIn) (vs. 1/34 other overlapping DE genes within smoking GWAS risk locus) (Table S6), representing a 3.5-fold increase of smoking GWAS risk genes. These results suggest NIC SST and SA share addiction-relevant gene pathways that involve neurogenesis and myelination.

### Parental effect of SA is associated with allelic imbalance of expression (AIE)

To ascertain the molecular mechanism of the parental effect of SA in F1 male rats, we first examined the role of ChrX genes. We found that SA-associated DE genes in NAc core were enriched for ChrX genes (FDR = 0.0004). However, most of these ChrX genes (52/67) showed a reduced expression in SA-inclined subgroup A (Table S5), as opposed that most SA-associated genes were upregulated in the NAc core. These ChrX genes thus unlikely play a major role in the parental effect of SA.

**Fig. 5:**
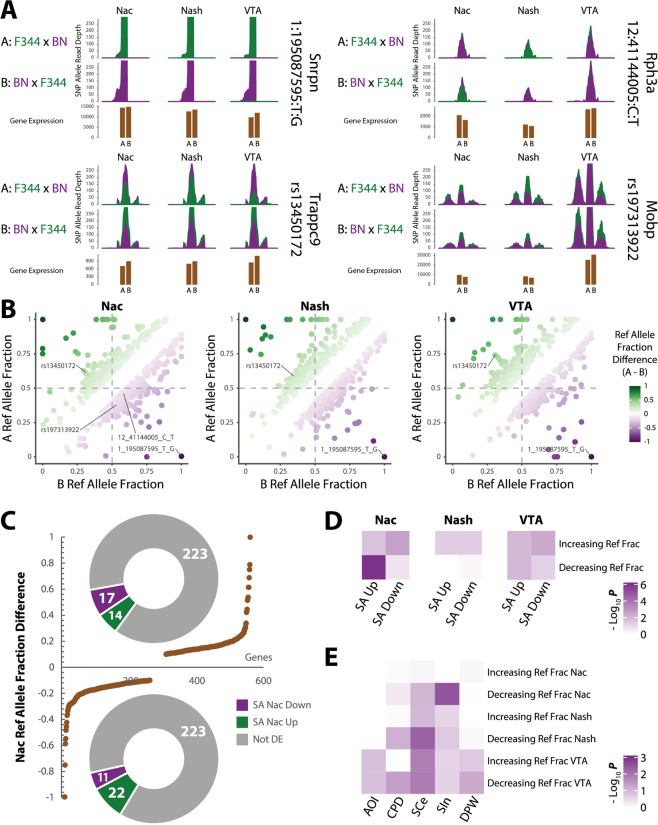
Allelic imbalance of expression (AIE) analysis of parental effect on self-administration (SA). **A** RNA-seq read pileup plots of example loci (transcribed SNPs) showing AIE in each brain region with a 500 bp window centered on the SNP. The sequencing reads of the Fischer-344 (F344) allele are in green and those from the Brown Norway (BN) allele are in purple. Upper row depicts read depth of AIE of A subgroup (SA inclined), middle row depicts read depth of AIE of B subgroup (SA disinclined), and lower row depicts the mean normalized gene expression (DESeq normalized mean RNA-seq read count) in A and B subgroups (shown in brown) for the SNP. **B** In each brain region (NAc core, shell and VTA; from left to right), correlation of the reference allele (BN copy) fraction of all AIE SNPs (binomial test, FDR < 0.05; ref allele fraction difference>0.1) between the subgroup A and B rats. **C** Distribution of the reference allele (BN copy) fraction differences (A subgroup–B subgroup) of AIE genes in the NAc core (brown line). Donut plots show the number of genes in each DE category (upregulated—green; downregulated—purple; no change—gray) associated with AIE SNPs that showed either increased (above, >0.1) or decreased (below,<-0.1) reference allele fraction in subgroup A vs. B. **D** Heatmap showing the Fisher’s exact test enrichment of up- or downregulated genes in NIC SA among genes showing differential AIE (increasing or decreasing reference allele fraction; 2-sample proportion test, FDR < 0.05) in subgroup A vs. B in each brain region. **E** Heatmap showing the Fisher’s exact test enrichment of genes harboring risk variants (*r*^2^ > 0.3 with GWAS index SNPs) of NIC addiction phenotypes among AIE genes with significantly increasing or decreasing reference allele fraction (2-sample proportion test, FDR < 0.05) in subgroup A vs. B in each brain region.

We then hypothesized that the SA-inclined subgroup A was due to the dominant paternal (F344 strain) allelic effect on SA-relevant genes, manifesting as differential AIE between the A and B subgroups. Using the transcribed heterozygous SNPs as proxies for regulatory variants, we compared the RNA-seq reads of the two parent alleles at a SNP site to identify AIE (see “Materials and methods”) (Fig. 5A). We found that among ~8000 transcribed bi-allelic SNPs, 47–55% showed either A or B subgroup AIE (FDR < 0.05, binomial test) (Fig. S10A, Tables S10, S11). A two-sample proportion test identified 719, 748, and 637 SNPs showing differential AIE (FDR < 0.05) between the A and B subgroups across NAc core, shell, and VTA regions, respectively. Although the reference allelic fractions of these AIE SNPs were correlated between the A and B subgroups (Pearson’s *R* ~0.24) (Fig. 5B and Table S10), >80% were region-specific (Fig. S10B, C).

To identify genes associated with the parental effect on SA, we first examined SNPs showing the largest difference of AIE (>70% allele fraction difference) (Fig. 5A, B). We found that such AIE SNPs were rare (*n* = 12) (Fig. S10D and Table S11), and are mostly in known imprinted genes: *Snrpn* [47, 48], *Ube3a* [49], and *Trappc9* [50]. AIE of *Snrpn* showed a stronger bias toward the paternal allele in subgroup B and was downregulated in the VTA of subgroup A (Fig. 5A, B and Table S11). *Trappc9* showed AIE bias toward the maternal SA-disinclined allele (BN) in subgroup A with the strongest bias in the VTA, which correspondingly showed downregulation in VTA (Fig. 5A, B and Table S11).

In the NAc core most transcriptionally relevant to SA, most genes showed small to modest AIE differences between subgroups A and B (Fig. 5C). However, we observed a strong enrichment (2.4-fold, Fisher’s exact test *p* < 2.1 × 10^−10^) of the SNPs with decreased AIE in the NAc core (Decreasing-Ref-Frac) among genes upregulated in NAc core of subgroup A (Fig. 5C, D). Of these 22 genes (Fig. 5C), 5 are associated with myelination (Fisher’s exact test, 30-fold, *p* < 6.2 × 10^−7^) and 3 within smoking GWAS risk loci (Fisher’s exact test, 4-fold, *p* < 0.04) (Table S1, Fig. S11A). STRING network analysis of these 22 genes (Fig. S11B, C) identified myelin sheath as the top-ranking enriched GO that includes *Serinc5*, *Cntn2*, *Mobp*, and *Thy1* (Fig. S11C, Table S13). NAc core genes with Decreasing-Ref-Frac also exhibited the strongest enrichment for GWAS associations for SIn in MAGMA analysis (Fisher’s exact test, FDR < 0.03) (Fig. 5E): the SIn-associated *Rabphilin 3A (Rph3A)* (Fig. 5A) encodes a protein that interacts with GluN2A and PSD-95 [9]. These results suggest a mechanistic role of strain-specific AIE of autosomal genes in NAc core and VTA of F1 rats in the observed imprinting of NIC SA.

## Discussion

Studies of the sex-specific NIC SST have yielded mixed results. For example, two previous reports show greater locomotor sensitization in female Sprague-Dawley rats [51, 52], while Pehrson et al. [53] report greater locomotor sensitization in male Sprague-Dawley rats. With F344/BN F1 rats, we found that only males showed NIC SST despite a strong locomotor response in females (Fig. 1D, E). Our transcriptomic analysis identified a strong negative correlation of NIC-induced expression changes between male and female VTAs (Fig. 3A). We also found that DE genes with opposite expression changes in male and female VTA likely contribute to the sex-specific NIC SST. The enriched GO-term, myelin sheath, among these VTA genes (Fig. 2E) also seems to be consistent with male-specific NIC SST: in rats, gestational exposure to NIC can produce sex-specific myelination effects during development [54, 55]; in humans, sex-specific myelination is also correlated with differential brain development in boys and girls [56, 57]. Thus, the identified transcriptomic signature of male-specific NIC SST in rat may provide mechanistic insight into why men tend to use tobacco at higher rates than women [58].

We have found an unexpected parental effect of NIC SA (Fig. 1F, G), which enabled us to identify the transcriptomic signatures of NIC SA by directly comparing the two reciprocally crossed subgroups. Both VTA and NAc core were found transcriptionally relevant to NIC SA. In aggregate, only AIE SNPs with Decreasing-Ref-Frac in NAc core of subgroup A rats were enriched for genes upregulated in NAc core (Fig. 5C, D), and for GWAS associations with SIn (Fig. 5E), suggesting region-specific AIE of autosomal genes may mechanistically explain the parental effect of NIC SA. Some genes with the strongest AIE differences between the A and B subgroups are known to be imprinted and likely contribute to the parental effect of NIC SA. For instance, *Snrpn*, a known paternally expressed gene [47, 48], and its bicistronic transcript partner *Snurf*, play an important role in adult neurogenesis [59]. Another known imprinted gene, *Trappc9*, also displayed AIE and was downregulated in subgroup A (Fig. 5A); interestingly, the paternally biased AIE SNPs of *Trappc9* reside in an intron (Fig. S12), whose corresponding human intron encompasses a paternally expressed *PEG13* that can silence the maternal transcription of *Trappc9* [50, 60]. Overall, our AIE analysis further supports NAc core to be an essential region for SA, in particular, its potential to be influenced by imprinting effects. Because AIE SNPs are proxies of transcription-regulating variants, future study to identify regulatory variants with differential allelic chromatin accessibility [61, 62] may shed light on the causal mechanism of parental effect on NIC SA.

For the first time, we have shown that the transcriptomic profiles of NIC SST and SA in rats can inform the GWAS findings of human smoking phenotypes [4–9]. Our MAGMA analysis showed that the downregulated genes in male VTA of SST model and a subset of AIE genes associated with SST in NAc core were significantly enriched for GWAS associations with SIn (Figs. 2A, 5E, and Table S3). Given a possible gateway “drug” role of NIC [2, 3], our results may also have implications for understanding the genetic etiology and neurobiology of other drugs of addiction. For instance, the expression changes of NIC SA-associated genes and cocaine SA-associated genes [63] were strongly correlated (*R*^2^ = 0.22 and 0.33 for NAc core and VTA, respectively) (Table S5 and Fig. S13). Notably, four (*Unc5b, Tnrc6a,Tmx2, and Arid4a*) of the 26 AUD GWAS risk genes [64, 65] were upregulated in NAc core of NIC SA rats (Fig. S13A). It is also noteworthy that *CTNNA2*, a DE gene in NIC SST and SA, is associated with not only SIn[9], but also alcohol, heroin, and methamphetamine dependence in Han Chinese [66].

Although our observations of male-specific NIC SST and the parental effects of NIC SA in male F344/BN F1 rats are interesting as discussed above, we acknowledge the limitations of our study. First and foremost, the greater acute responding to NIC, but the lack of SST to NIC, in the F1 females in the present experiments may be a specific attribute of these rat strains. This is because gonadal hormones during the estrous cycle have been suggested to contribute to greater stimulant [41] and NIC [40] induced locomotion and locomotor SST in outbred females relative to males. We have initially chosen to study F344/BN F1 rats, because we aimed to explore the molecular mechanism of any observed imprinting effect on animal behaviors by examining AIE, in which the allelic imbalance analysis would benefit from phenotypic differences between two parental lines and in this case F344 is known to have the lowest sensitivity to NIC among six tested strains including BN [67]. As such, future experiments will need to explore whether F1 rats of some other strains would behave differently. Another limitation is that because of the lack of NIC SST in females, we only assayed males in the NIC SA paradigm. Given that female rats tend to display higher intake than males as shown in a recent meta-analysis [68], it may have proven intriguing to analyze the behavioral and transcriptomic data of NIC SA of these female F1 rats. Despite our observation that ChrX genes unlikely play a major role in the parental effect of SA in males, the female rats may exhibit sex-specific parental effects of NIC SA because of the well-known ChrX inactivation in females. However, we expect males and females will share some of the observed parental effects manifested as autosomal AIE, such as the paternally biased AIE SNPs of a Chr7 Trappc9 (Fig. S12), which harbors lncRNA (long noncoding RNA) Peg13, a known strong imprinting gene conserved between mice and humans [69]. Thus, more extensive examination of the behavioral and molecular changes observed in the brains of these F1 females following exposure to NIC, and their comparison to the changes observed in outbred wild-type female rats, may provide a better understanding of reported sex differences in liability to substance abuse.

In summary, our transcriptomic analyses suggested plausible mechanisms for the sex-specific NIC SST and the parental effect of NIC SA. Importantly, DE genes of SST and SA are enriched for GWAS risk SNPs of smoking phenotypes. However, in light of the cellular heterogeneity of each brain region, our conclusion, although interesting, is limited by the use of bulk brain RNA-seq, as opposed to single-cell RNA-seq (scRNA-seq) or single-nucleus RNA-seq (snRNA-seq), in our transcriptomic analyses. Future studies using scRNA-seq or snRNA-seq will allow for better characterization of which neural subtypes are involved in the sex-specific NIC SST and the parental effect of NIC SA, and the extent to which these differ across brain regions. Nonetheless, the current study advanced our mechanistic understanding of NIC addiction in rodents, providing a valuable resource for prioritizing the biologically relevant risk genes of smoking behaviors and for other drugs of abuse in humans.

## Funding and disclosure

This study was supported by the National Institutes of Health (NIH) grant DA041600 (to JD). The authors declare no conflict of interests.

## Supplementary information

Supplementary Table 1

Supplementary Table 2

Supplementary Table 5

Supplementary Tables 3,4,6-13

Supplemental Figures & Legends

## Data Availability

All code used to generate results for this analysis is available at: https://rbutleriii.github.io/center_for_psychiatric_genomics/. Sequence data and raw count matrices have been submitted to the Gene Expression Omnibus for SST and SA experiments, GSE157726 and GSE157683, respectively.
