# Supplementary material for: Sex-specific nicotine sensitization and imprinting of self-administration in rats inform GWAS findings on human addiction phenotypes
Source: Neuropsychopharmacology. 2021 May 18;46(10):1746–56. doi: 10.1038/s41386-021-01027-0 (PMC8358005; doi:10.1038/s41386-021-01027-0)
Supplement: Supplementary file 5 — Supplemental Figures & Legends [file 41386_2021_1027_MOESM5_ESM.docx]

Supplementary Materials

Index

1. Supplementary Figures S1 to S13…………………………………………2-17

1. Supplementary Tables S1 to S13 (only table titles are listed here; individual tables are provided as Datasets)…………………………………………………..18


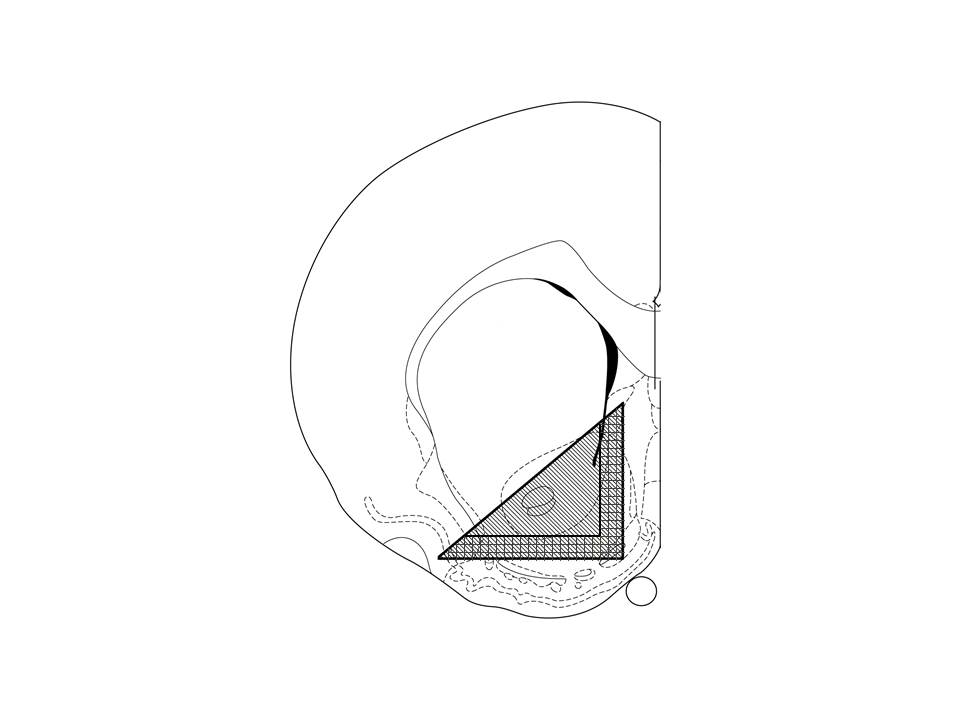


Fig. S1 Illustration of the NAc core and shell subregions dissected bilaterally for the transcriptomic experiments. NAc core is represented as diagonal hatched lines and NAc shell is

shown with cross hatched lines. Line drawing depicts the caudal surface of a coronal hemisection extending 1.2–2.2 mm from bregma and is from (Paxinos and Watson, 1977).


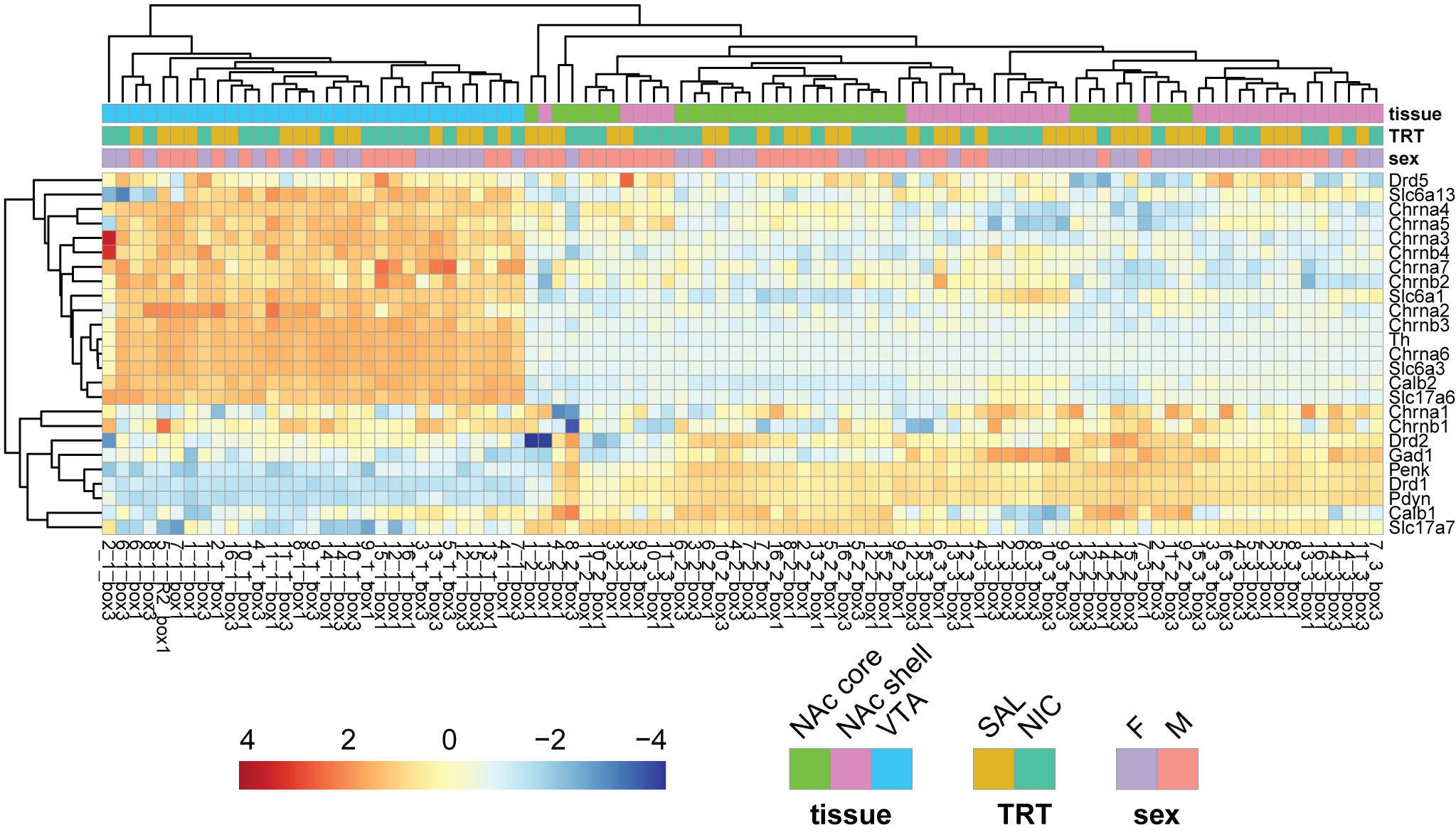


**Fig. S2** **RNA-seq sample clustering based on a set of brain region-specific genes.** Samples clustered by row-centered, variance stabilizing transform normalized values. Annotated by tissue (NAc core – nucleus accumbens core, NAc shell – nucleus accumbens shell, VTA – ventral tegmental area), treatment (TRT, NIC – nicotine injections, SAL – saline injections) and sex.


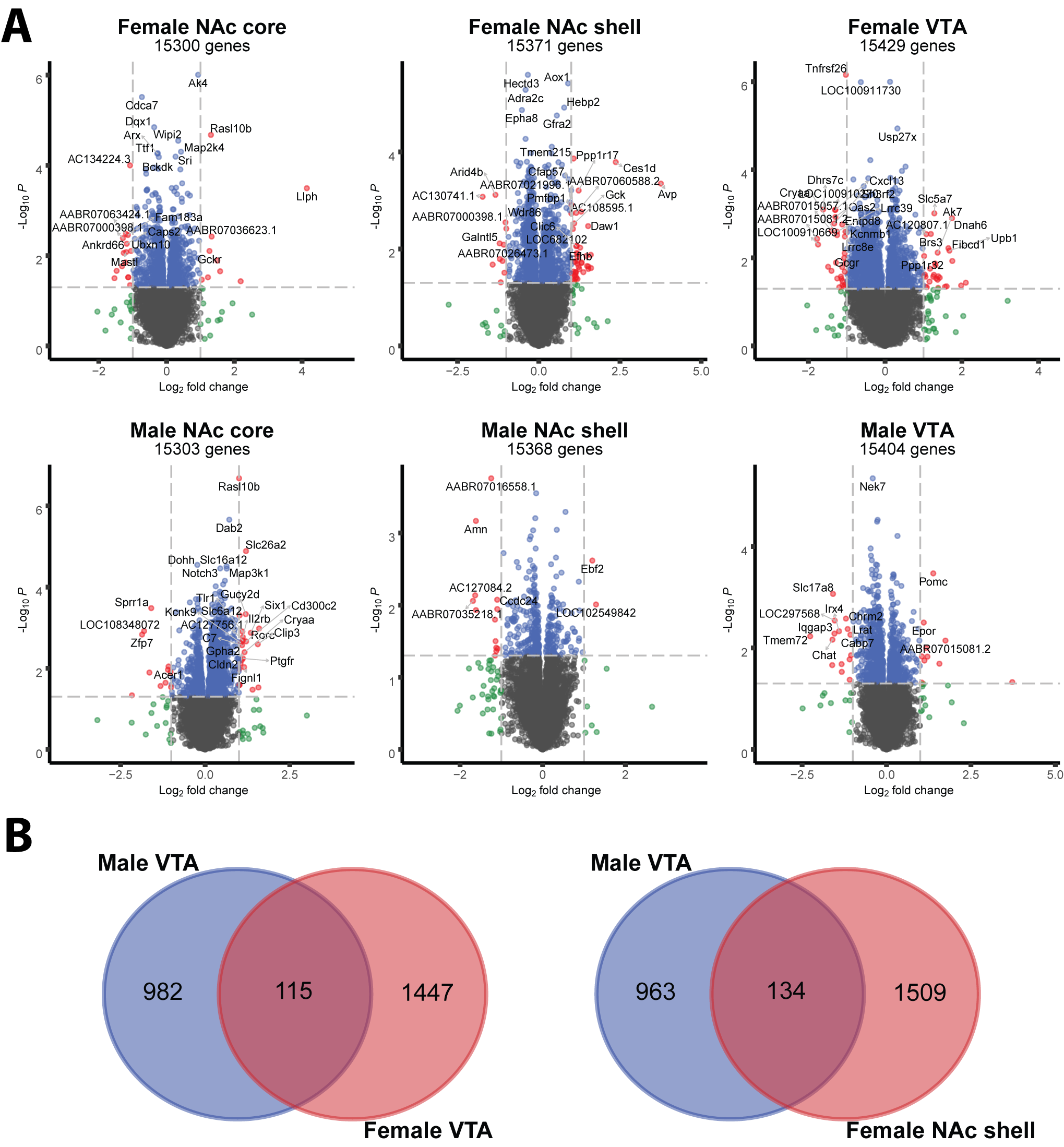


**Fig. S3** **Differential expression (DE) of genes in each brain region of NIC-treated vs control F1 rats.** (**A**) Volcano plots of DE genes in each brain tissue by sex. The horizontal dashed lines indicate 2-fold changes and the vertical dashed lines indicate the nominal significance *p*-value of 0.05. (**B**) Venn diagram showing the overlapping DE genes associated with NIC SST between males and females in regions transcriptionally relevant to tobacco use (determined from DAVID gene set enrichment analysis in Fig. 2E).


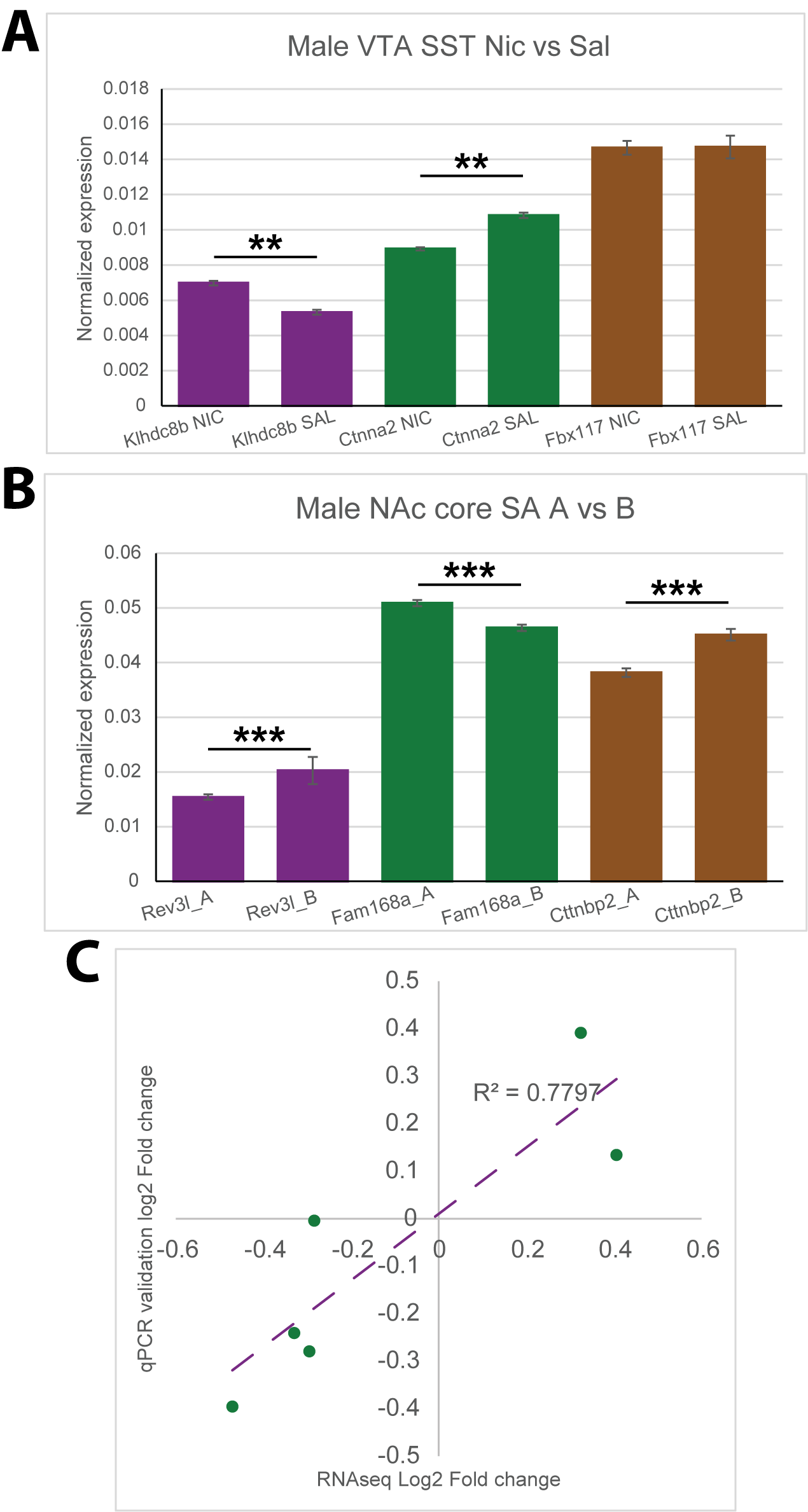


**Fig. S4 qPCR validation of the expression changes of 6 NIC SST- or SA-associated genes selected from RNA-seq DE analysis.** (**A**) Two DE genes associated with SST (*Klhdc8b*, *Ctnna2*; ** *p*<0.03) and (**B**) three genes associated with SA (*Rev3l*, *Fam168a*, *Cttnbp2*; *** *p*<0.02) were confirmed by qPCR. The expression change of *Fbxl17* in SST was not confirmed. Expression of each gene was normalized to *GAPDH*. Student’s *t*-test was used to test DE. Data are from 7 biological replicates (different animals) each with 3 technical replicates. Error bars, standard error of mean (SEM). **(C)** Strong correlation (R^2^=0.78) of gene expression fold changes of the selected genes between RNA-seq and qPCR validation.


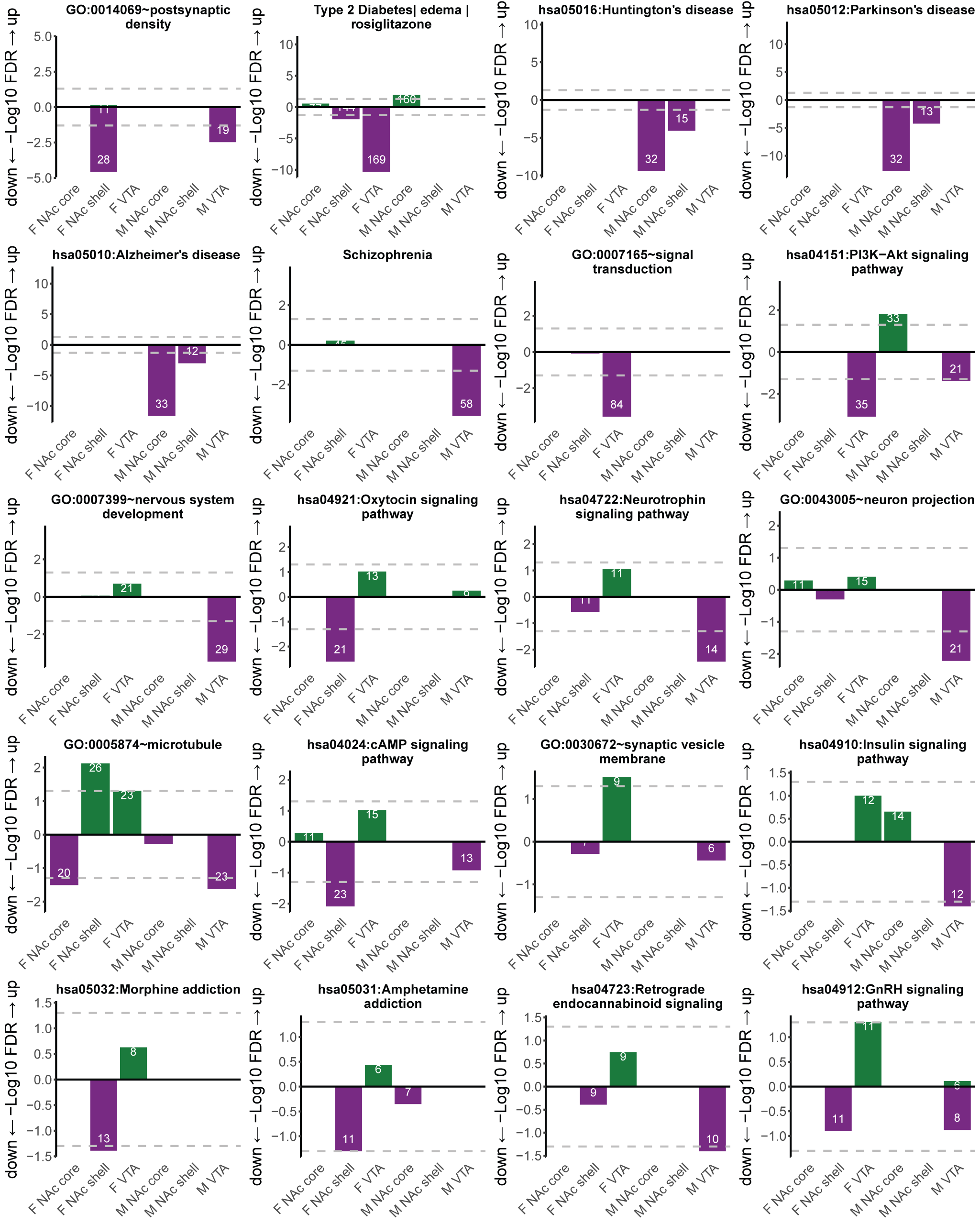


**Fig. S5 Extended DAVID gene set enrichment analysis of DE genes in each brain region for NIC sensitization.** Listed are for GAD diseases and disease classes, OMIM diseases, KEGG pathways and GO terms. FDR-significant gene sets include the number of class genes (inset in the bar).


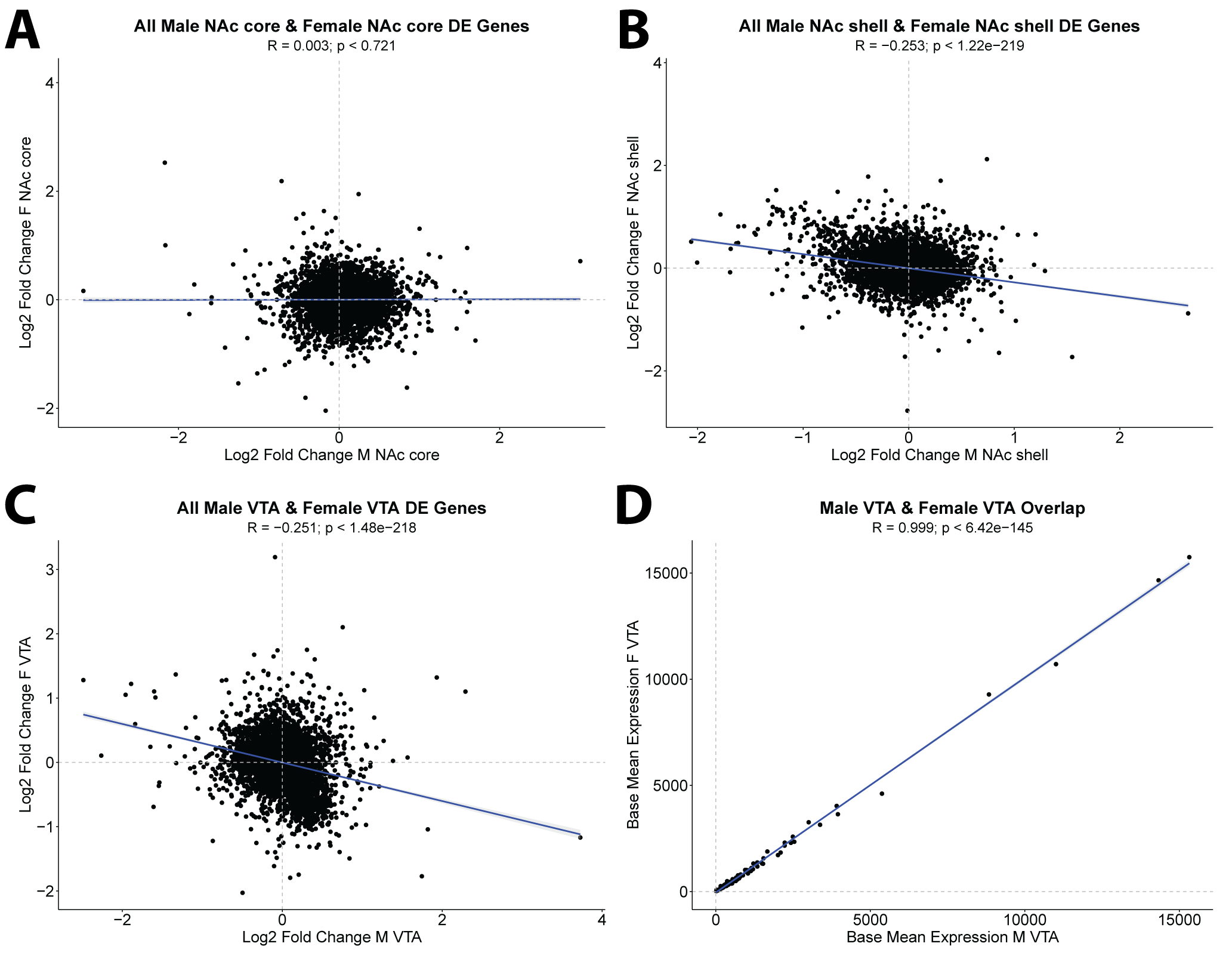


**Fig. S6 Transcriptome-wide correlation of gene expression fold-change between females and males in each brain region**. (**A**) Log2-fold change in NAc core, (**B**) Log2-fold change in NAc shell, and (**C**) Log2-fold change in VTA. (**D**) Correlation of base mean expression (DESeq normalized count) in the male and female VTA for DE genes (with *p*<0.05 as cut-off).


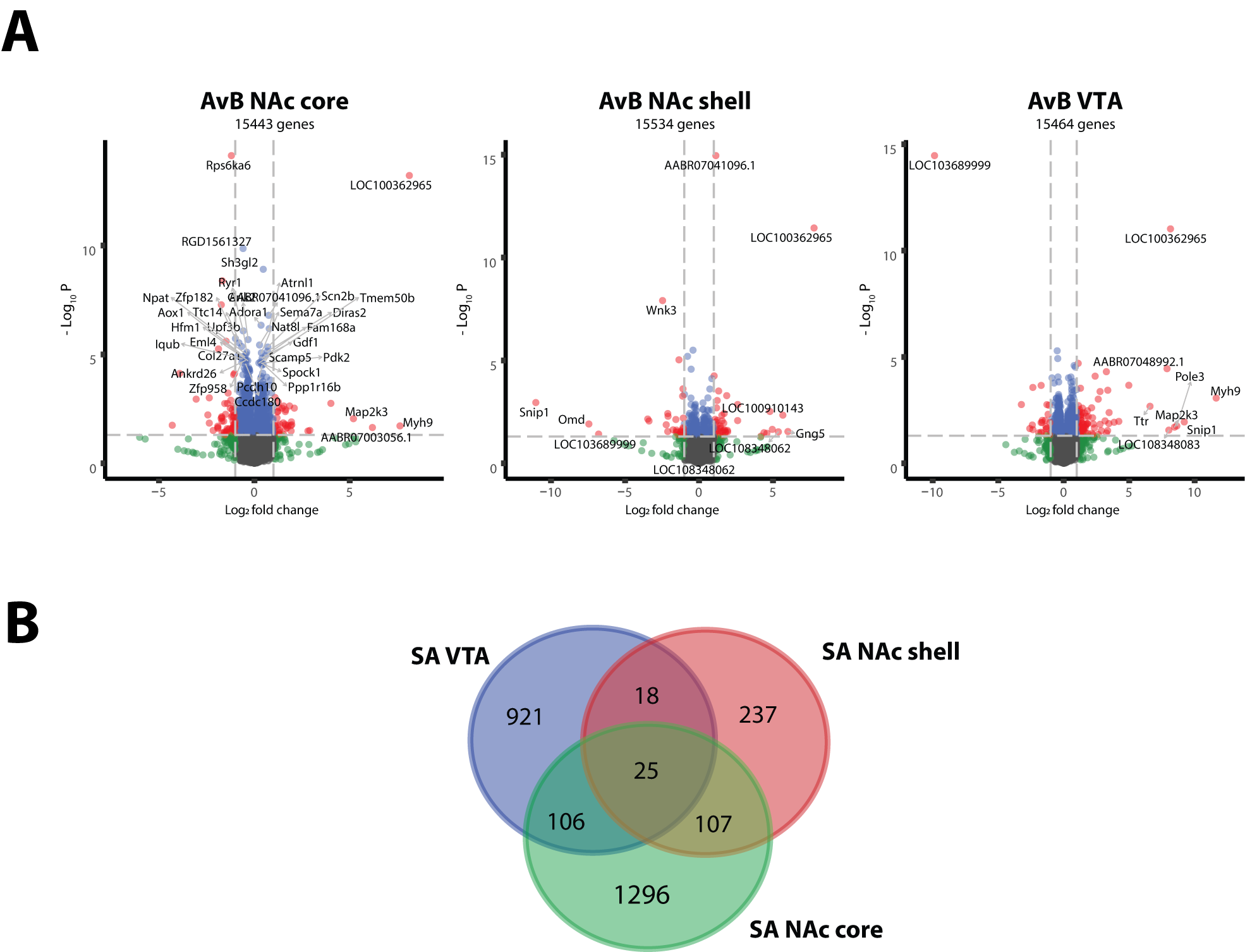


**Fig. S7 Differential expression (DE) of genes in tissues of self-administration A subgroup (SA-inclined) vs B subgroup (SA-disinclined) animals**. (**A**) Volcano plots of DE genes in each brain tissue. The horizontal dashed lines indicate 2-fold changes and the vertical dashed lines indicate the nominal significance *p*-value of 0.05. (**B**) Venn diagram showing the overlapping DE (*p*<0.05) genes associated with NIC SA between different brain regions.


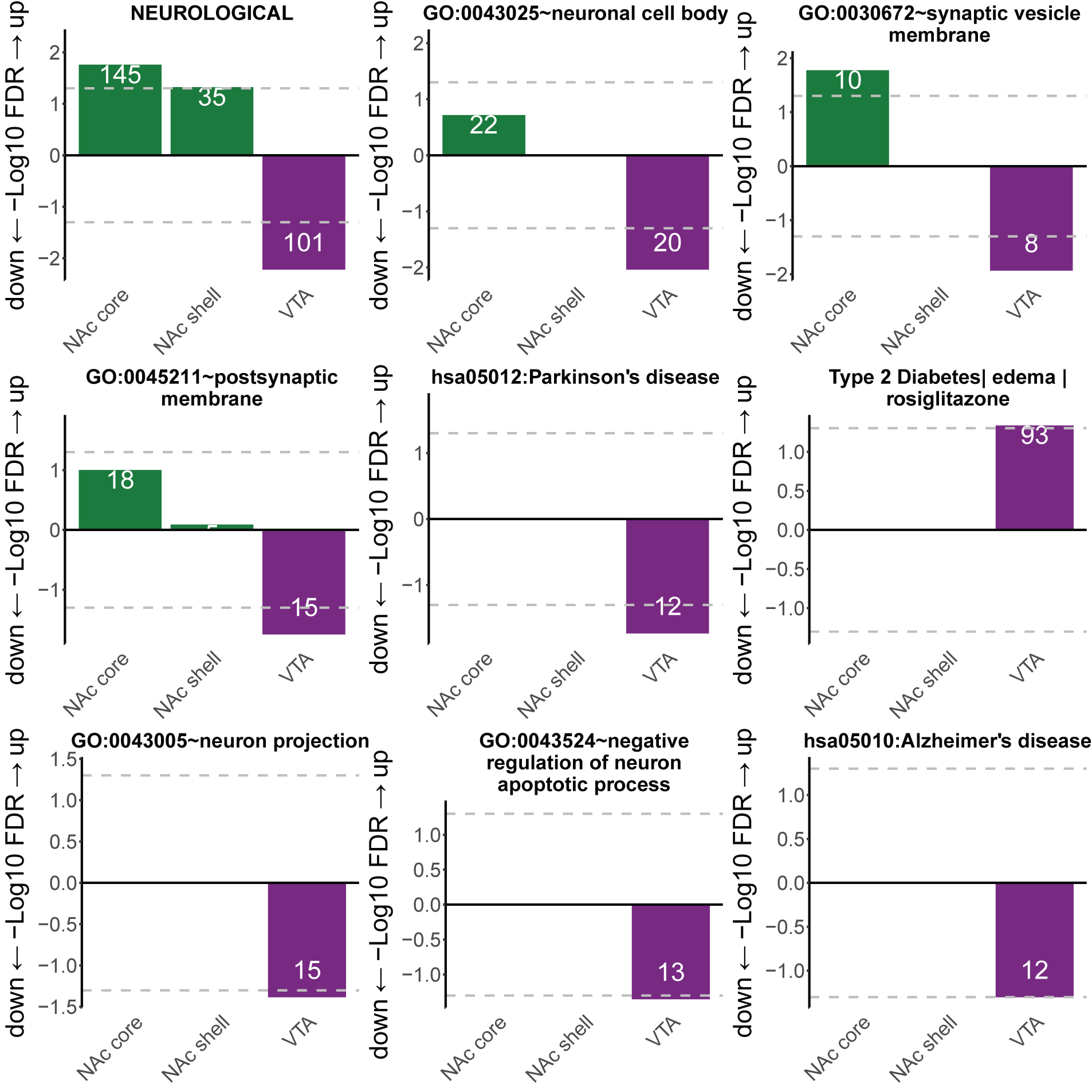


**Fig. S8 Extended DAVID gene set enrichment analysis of DE genes in each brain region for NIC self-administration.** Listed are for GAD diseases and disease classes, OMIM diseases, KEGG pathways and GO terms. FDR-significant gene sets include the number of class genes (inset in the bar).


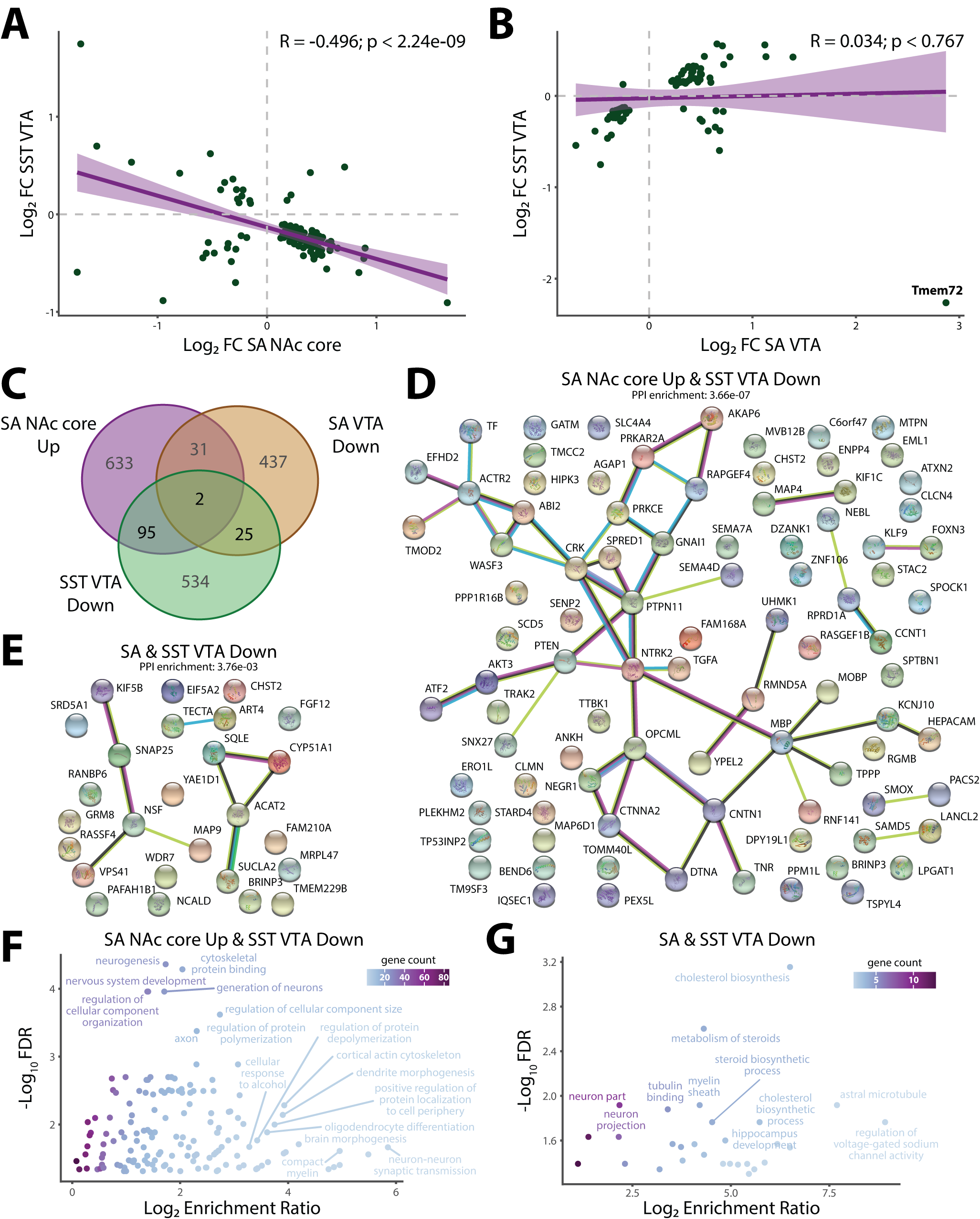


**Fig. S9 Comparison of differential expression (DE) of genes in regions enriched for addiction phenotypes between NIC sensitization (SST) and self-administration (SA)**. (**A**) Fold change (FC) comparison of genes with nominally significant (*p*<0.05) DE in the SST ventral tegmental area (VTA) and the SA NAc core (all from male rats). (**B**) FC comparison of genes with nominally significant (*p*<0.05) DE in the SST male VTA and the SA VTA. Note: excluding *Tmem72* (labeled), an expression outlier, gave a significantly positive correlation (R=0.66 and *p*<1.2×10^-10^). (**C**) Venn diagram of nominally significant (*p*<0.05) DE genes in directions/regions enriched for addiction phenotypes. Note: SST male VTA and SA VTA downregulated, as well as SA NAc core upregulated genes are most relevant to NIC addiction based on DAVID enrichment analyses (Figs. 3, 5, Fig. S6). (**D**) STRING analysis of the human orthologs of 97 genes with opposite DE direction in SA VTA (downregulated) and SA NAc core (upregulated). Number of nodes: 91, number of edges: 51, average node degree: 1.12, avg. local clustering coefficient: 0.333, expected number of edges: 23, PPI enrichment *p*<3.7×10^-7^. (**E**) STRING analysis of the human orthologs of 27 genes with concordant DE direction (downregulated) in SA VTA and SST male VTA. Number of nodes: 26, number of edges: 9, average node degree: 0.692, avg. local clustering coefficient: 0.321, expected number of edges: 3, PPI enrichment *p*<3.8×10^-3^. (**F, G**) Ontological enrichments from the STRING analysis in (**E**) and (**D**), respectively, colored by gene count in each term.


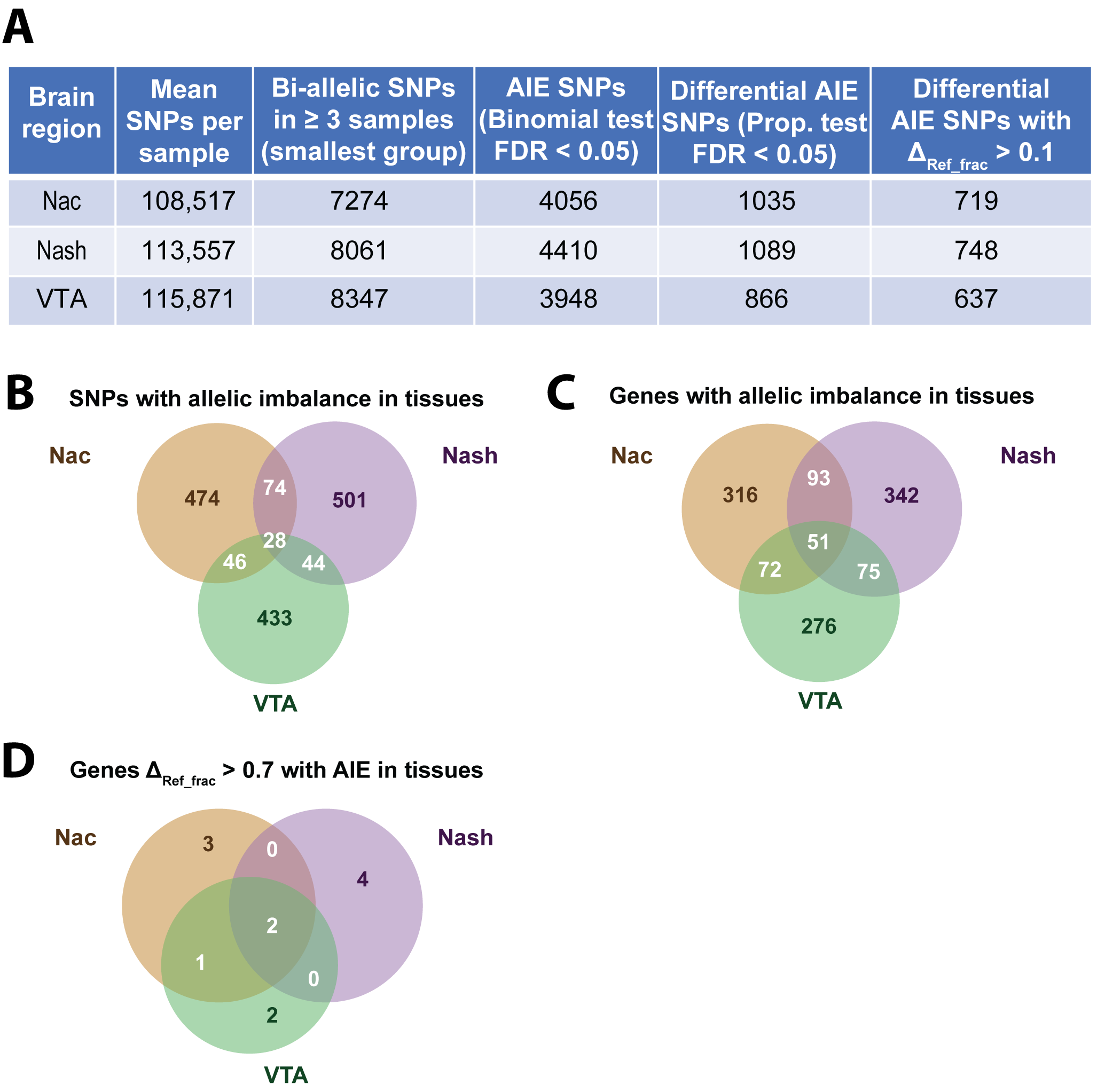


**Fig. S10 Allelic imbalance of expression (AIE) statistics in self-administration A subgroup (SA-inclined) and B subgroup (SA-disinclined) animals**. (**A**) Total number of AIE SNPs at different cut-offs in each brain region. (**B**) Venn diagram of AIE SNPs in each brain region. (**C**) Venn diagram of genes containing AIE SNPs in each brain region. (**D**) Genes containing AIE SNPs with a relatively large difference of AIE between subgroups A and B (Reference allele fraction difference between subgroups>0.7).

**Fig. S11 Allelic imbalance of expression (AIE) in genes with upregulated expression in NAc during self-administration (SA).** (**A**) RNA-seq read pileup plots of example loci (transcribed SNPs) showing AIE in each brain region. The sequencing reads of the Fischer-344 (F344) allele are in green and those from the Brown Norway (BN) allele are in purple. Upper row depicts AIE of A subgroup (SA inclined), middle row depicts AIE of B subgroup (SA disinclined), and lower row depicts the normalized gene expression (mean RNA-seq read count) in A and B subgroups (shown in brown). (**B**) STRING analysis of human orthologs of the 22 genes with AIE and DE combined in the NAc core; with decreasing reference allele fraction in subgroup A and upregulated expression during SA (*p*<0.05). Number of nodes: 22, number of edges: 5, average node degree: 0.455, avg. local clustering coefficient: 0.364, expected number of edges: 1, PPI enrichment *p*<6.0×10^-3^. (**C**) Gene ontological enrichments from the STRING analysis in (**B**) colored by gene count in each term. X-axis indicates the Log2 enrichment ratio and Y-axis indicates the -Log10 FDR.


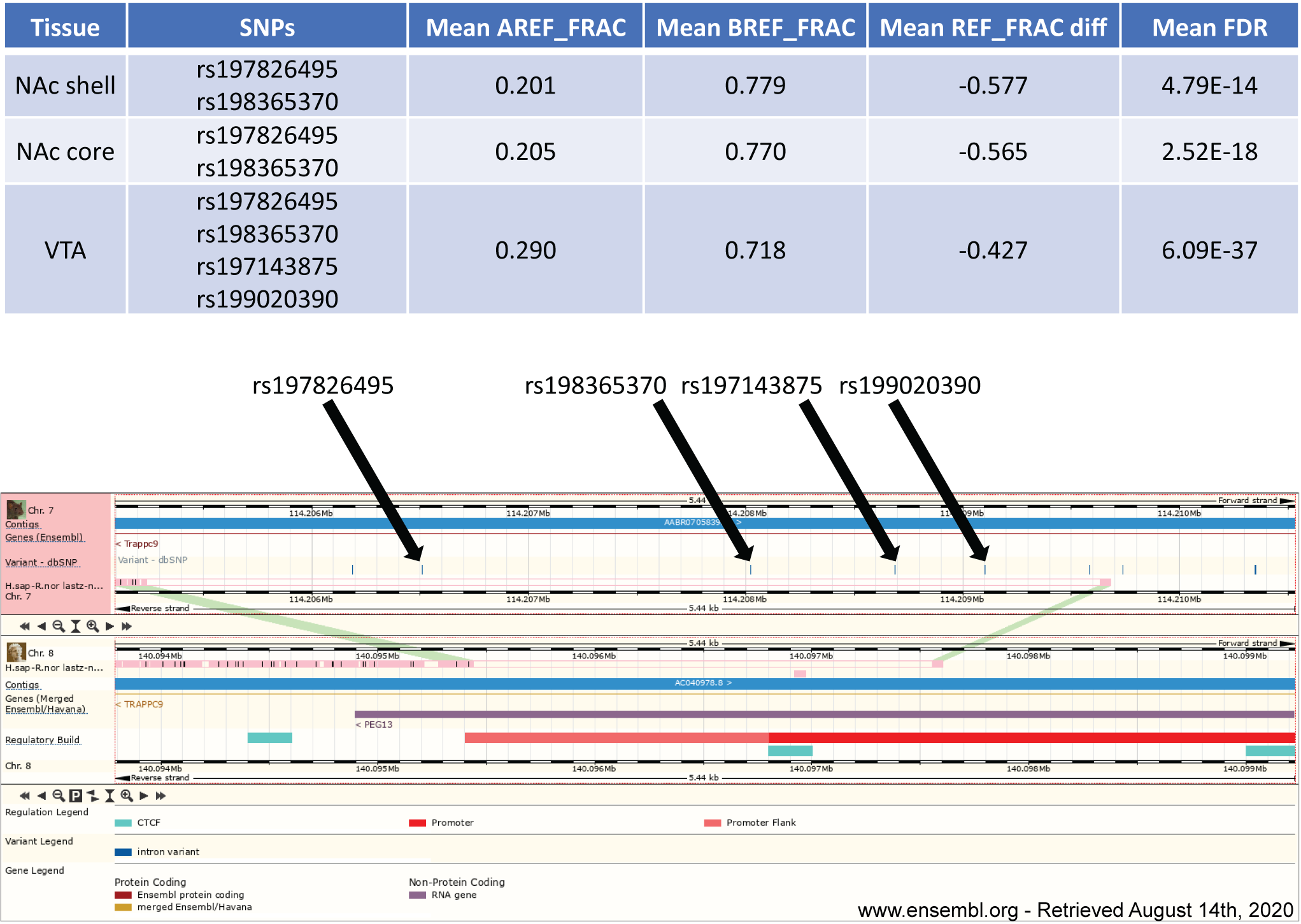


**Fig. S12** **Intronic allelic imbalance of expression (AIE) SNPs in *Trappc9*, aligned to putative site of *paternally expressed gene 13* (*PEG13*) rat ortholog**. Four transcribed SNPs showing strong AIE (upper table) in the intronic region of *Trappc9* identified in a rat (Rnor_6.0) to human (GRCh38.p13) LASTz alignment (lower; light pink), via ensemble.org. Rat *Trappc9* intron is shown in maroon in the rat genome, Human *PEG13* and *TRAPPC9* are shown in purple and yellow, respectively, on the human genome.


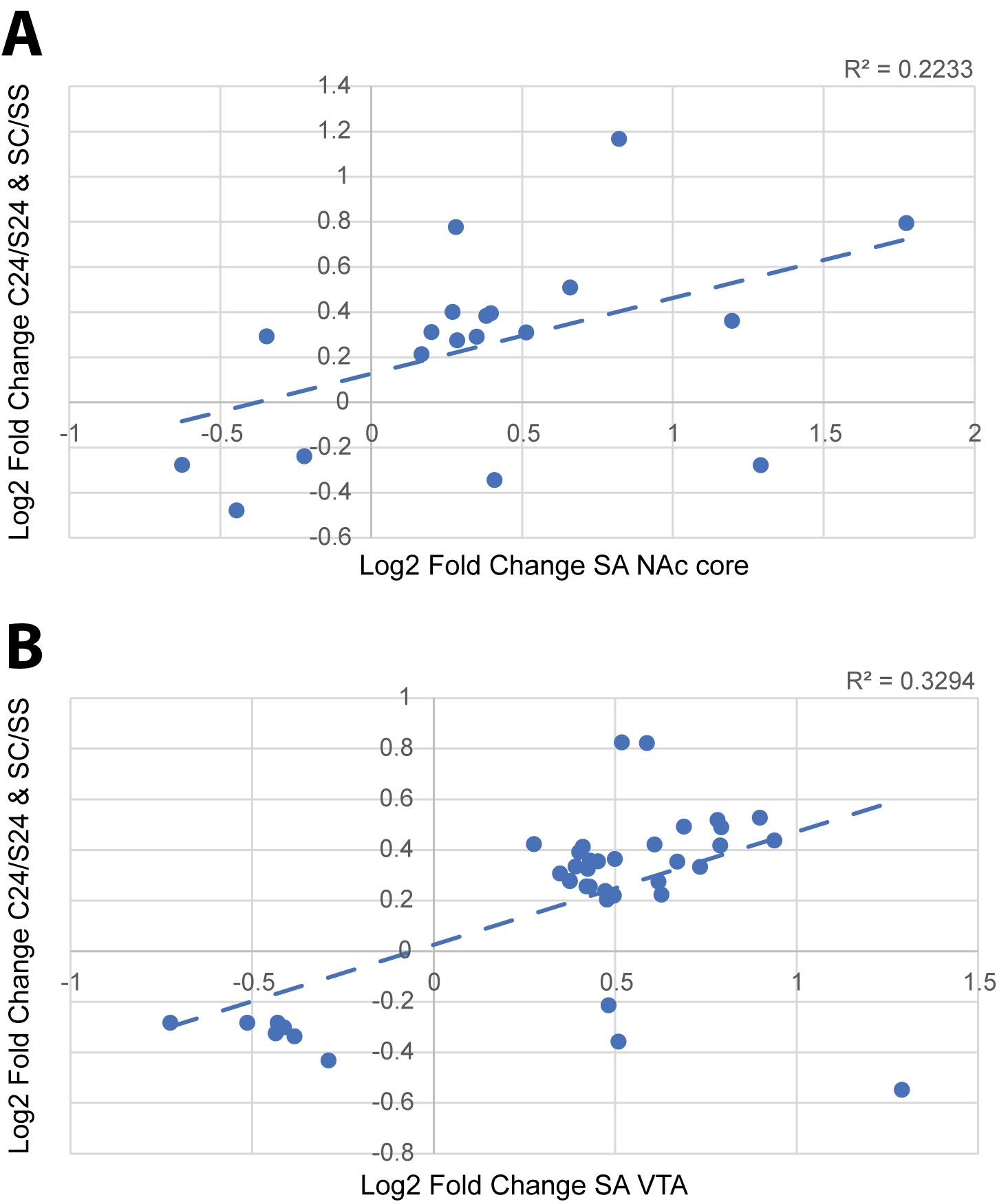


**Fig. S13** **Correlation between self-administration (SA) gene differential expression (DE) and cocaine addiction gene DE in Walker et al 2018**. (**A**) DE of SA nucleus accumbens (NAc) core genes that are also significantly DE in cocaine SA + 24-hour withdrawal (C24) vs saline SA + 24-hour withdrawal (S24) and saline SA + 30-day withdrawal + cocaine exposure (SC) vs saline SA + 30-day withdrawal + saline exposure (SS). (**B**) DE of SA ventral tegmental area (VTA) genes that are also significantly DE in C24 vs S24 and SC vs SS.

**Supplementary Tables (Only titles are listed here; Tables are provided as DataSets)**

Table S1. Genes differentially expressed in male sensitized rats.

Table S2. Genes differentially expressed in female sensitized rats.

Table S3. Top-ranking sensitization (SST) VTA and self-administration (SA) NAc core DE genes associated with smoking GWAS phenotypes

Table S4. Overlapping genes associated with NIC SST between male and female VTA.

Table S5. Genes differentially expressed in self-administration inclined rats.

Table S6. Overlapping genes between SA NAc core and SST VTA.

Table S7. Overlapping genes between SA VTA and SST VTA males.

Table S8. STRING Protein Enrichment of 27 Gene Overlap Between SA VTA down and SST VTA down.

Table S9. STRING Protein Enrichment of 97 Gene Overlap Between SA Nac core up and SST VTA down.

Table S10. Allelic Imbalance of expression SNPs showing a parental effect.

Table S11. Allelic Imbalance of expression genes showing a parental effect.

Table S12. Literature search of 22 gene overlap between SA NAc core up and AIE NAc core decreasing ref fraction.

Table S13. STRING protein enrichment of 22 gene overlap between SA NAc core up and AIE NAc core decreasing ref fraction.
